# Lost in the shadows: reflections on the dark side of co-production

**DOI:** 10.1186/s12961-020-00558-0

**Published:** 2020-05-07

**Authors:** Oli Williams, Sophie Sarre, Stan Constantina Papoulias, Sarah Knowles, Glenn Robert, Peter Beresford, Diana Rose, Sarah Carr, Meerat Kaur, Victoria J. Palmer

**Affiliations:** 1 Florence Nightingale Faculty of Nursing, Midwifery & Palliative Care, King’s College London, 4th Floor, James Clerk Maxwell Building, 57 Waterloo Road, London, SE1 8WA United Kingdom; 2 THIS Institute, Cambridge, United Kingdom; 3 Service User Research Enterprise, King’s College London, London, United Kingdom; 4 University of York, York, United Kingdom; 5 University of Essex, Colchester, United Kingdom; 6 University of Birmingham, Birmingham, United Kingdom; 7 NIHR ARC Northwest London, London, United Kingdom; 8 The University of Melbourne, Melbourne, Australia

**Keywords:** Co-production, collaboration, participatory research, collaborative research, applied health research, research impact, dark logic, unintended consequences, user involvement, patient and public involvement

## Abstract

This article is a response to Oliver et al.’s Commentary ‘The dark side of coproduction: do the costs outweigh the benefits for health research?’ recently published in *Health Research Policy and Systems* (2019, 17:33). The original commentary raises some important questions about how and when to co-produce health research, including highlighting various professional costs to those involved. However, we identify four related limitations in their inquiry, as follows: (1) the adoption of a problematically expansive definition of co-production that fails to acknowledge key features that distinguish co-production from broader collaboration; (2) a strong focus on technocratic rationales for co-producing research and a relative neglect of democratic rationales; (3) the transposition of legitimate concerns relating to collaboration between researchers and practitioners onto work with patients, service users and marginalised citizens; and (4) the presentation of bad *practice* as an inherent flaw, or indeed ‘dark side’, of co-production without attending to the corrupting influence of *contextual* factors within academic research that facilitate and even promote such malpractice. The Commentary’s limitations can be seen to reflect the contemporary use of the term ‘co-production’ more broadly. We describe this phenomenon as ‘cobiquity’ – an apparent appetite for participatory research practice and increased emphasis on partnership working, in combination with the related emergence of a plethora of ‘co’ words, promoting a conflation of meanings and practices from different collaborative traditions. This phenomenon commonly leads to a misappropriation of the term ‘co-production’. Our main motivation is to address this imprecision and the detrimental impact it has on efforts to enable co-production with marginalised and disadvantaged groups. We conclude that Oliver et al. stray too close to ‘the problem’ of ‘co-production’ seeing only the dark side rather than what is casting the shadows. We warn against such a restricted view and argue for greater scrutiny of the structural factors that largely explain academia’s failure to accommodate and promote the egalitarian and utilitarian potential of co-produced research.

“*Synergetic outcomes can be fostered to a much greater extent than our academic barriers have let us contemplate*.” Elinor Ostrom ([1], p. 1083, emphasis added)

## ‘Cobiquity’: what is lost when any form of collaboration becomes ‘co-production’?

In a recent article, Oliver et al. [2] encourage consideration of a number of important questions related to co-production in research, in particular, under what circumstances should co-production be advocated rather than other (e.g. consultative) approaches, and what types of infrastructure are needed to support *productive coproduction*? These are important questions for researchers, organisations, citizens and funders to consider before promoting or undertaking participatory research of any kind. As researchers in this field, we welcome the authors’ call for greater recognition of the skilled nature of such work, their sympathetic discussion of the personal costs experienced by some professionals involved, and their willingness to identify inequities in labour and reward. However, Oliver et al. [2] pursue these useful lines of inquiry with a problematically expansive definition of co-production, which is simultaneously restricted by largely technocratic and instrumental rationales for co-producing research. We challenge these views by drawing on the foundational work – and subsequent critiques – that underpin the concept of co-production and its evolution since the early 1970s. Our main concerns are how the limitations of Oliver et al.’s [2] critique neglects the unique and vital perspectives of patients, service users and marginalised citizens, and how this may have a detrimental impact on efforts to enable co-production with marginalised and disadvantaged groups. Additionally, we advocate for greater consideration of the structural inequalities in academia and beyond that impede co-production.

The context for the original article – and our response – is a “*participatory zeitgeist*”, defined by a confluence of social, cultural and political change leading to increasing interest in methods for citizen engagement, public participation and involving people with relevant lived experience in the tasks of health system (re)design and service improvement [3]. Oliver et al. [2] acknowledge this by stating that there are “*many forms of collaborative research practices*” and a “*multiplicity of modes by which researchers may interact with stakeholders*”. However, the broad use of ‘stakeholders’ throughout their Commentary does not, in our view, sufficiently distinguish between service users, public contributors and professionals (e.g. healthcare practitioners, commissioners, policy-makers and industry partners). Such distinctions implicate very different types of collaborative work, based on differences in expertise, experience and power, and we contest whether the latter (partnerships exclusively between or primarily led by researchers and professionals) should be referred to as co-production.

Oliver et al.’s [2] working definition of co-production as “*the joint working of people who are not in the same organisation to produce goods or services*” originates from the work of Ostrom [1]. The expansive nature of this definition facilitates an interpretation that almost any service or product development that occurs between people who are not formal work colleagues is, by definition, co-produced. The utility of this definition was questioned over a decade ago when it was argued that “*partnership is now so normal in services as to render such definitions trivial*” ([4], p. 847). Oliver et al. [2] are certainly not alone in employing a broad definition of co-production. British think tanks confused the matter early in the twenty-first century by essentially creating a “*hybrid co-production discourse*” by using the *language* of radical power sharing to promote entrepreneurial government [5]. Therefore, Oliver et al.’s [2] working definition to some extent simply reflects the reality of the term’s contemporary use by funders, policy-makers and researchers. However, such usage represents a phenomenon that we term ‘cobiquity’ – an apparent appetite for participatory research practice and increased emphasis on partnership working, in combination with the related emergence of a plethora of ‘co’ words, promoting a conflation of meanings and practices from different collaborative traditions. This phenomenon commonly leads to a misappropriation of the term ‘co-production’ as the conflation disregards significant differences between collaborative traditions, such as who is involved, how they are involved, the experiences people bring, and to what extent such processes address structural and interpersonal inequalities in power.

Notably, Ostrom went on to clarify that co-production “*implies that citizens can play an active role in producing public goods and services of consequence to them*” ([1], p. 1073). However, while some passing references to patient and public involvement are made, consideration of the centrality of patients, service users and citizens to the theory and praxis of co-production is almost entirely absent in Oliver et al.’s [2] critique. Neglecting this fundamental aspect may account for the peer-reviewer’s negative response to the initial draft, commenting that it did “*a disservice to those who are working hard to fully engage in hearing the patient voice, and allowing that voice a place at the research table*” ([6], p. 2). This oversight could have been avoided if the Commentary more fully appreciated the various rationales for co-producing research. Instead, by defining co-production as partnership working between people in different organisations, Oliver et al. [2] muddy the waters – their critique inaccurately transposes concerns that quite legitimately relate to inter-organisational collaboration between researchers and practitioners onto work with patients, service users and marginalised citizens. Addressing this imprecision is one of our main motivations for writing this response.

Oliver et al.’s [2] adoption of a working definition of co-production, which essentially merely describes *collaboration*, may explain why the National Institute for Health Research’s Collaborations for Leadership in Applied Health Research and Care (NIHR CLAHRCs) are their example of a “*truly coproductive*” approach – especially given how common it is for researchers to describe applied health research in this way [7, 8]. This is particularly revealing when paired with their assertion that often “*a financial contribution by the stakeholder is indicative of an authentic coproduction partnership*” ([2], p. 3). Few would regard the CLAHRC network as a paragon of co-produced research, although it is certainly recognised for having had a remit for collaboration (considered novel when the network was conceived in 2008) to address the long-standing translational gap between health(care) research and practice [9]. The surprising notion that a financial contribution from the ‘stakeholder’ is required to confirm the authenticity of a co-production partnership demonstrates that Oliver et al. [2] have relatively powerful (e.g. industry) partners in mind. This frames co-production as primarily a potential means for researchers to work with practitioners to ensure research addresses the central concerns of service providers rather than, for instance, for affording patients, services users and/or marginalised citizens more power to address their needs and concerns. Again, Oliver et al. are not alone in addressing co-production in research as primarily an endeavour for bringing together researchers and ‘practitioners’ [10]. Such an approach may increase the likelihood of service providers implementing research findings and therefore be important in a ‘knowledge into action’ model [11]. However, it stands in rather stark contrast to the egalitarian tradition more commonly aligned with co-production through Ostrom’s seminal work [12–14], patient and community activism [15–19], and Cahn’s conceptualisation [20], which more explicitly locates power and worth with citizens in order to address issues of social justice.

While collaborative (as opposed to co-produced) research may increase knowledge translation and uptake, it does not necessarily share the aim of making the conception or delivery of such research or services – or indeed the design process – more egalitarian, democratic or transparent. The phenomenon of cobiquity leads to these critical elements of co-production being neglected, for example, consideration of the role of power and the goal of enacting relationships that (unlike traditional research collaborations) address the needs of patients, service users and/or marginalised citizens, in part through ascribing legitimacy to ‘lay’ knowledge [20–24]. This is not to claim that Oliver et al. [2] are unaware of the ethical and political underpinnings commonly considered essential to the theory and praxis of co-production – these are briefly acknowledged in their Commentary – but rather to emphasise that their adopted definition and technocratic priorities allow for, and could even be considered to promote, ‘co-produced’ research with marginal roles for service users, patients and/or marginalised citizens or research without them entirely. For instance, the focus on utilising co-production to make research “*implementation ready*” leads Oliver et al. to argue that “*there may be alternative ways to achieve this without risking the costs* [of co-production]” ([2], p. 8) without acknowledging that alternatives are liable to reduce the focus on inclusivity and equity (an outcome we regard as a ‘cost’).

## Bringing rationales for co-production to the fore

For clarity, we are not indulging in mere semantic quibbling or endeavouring to draw “*clear lines along blurred boundaries*” as a form of “*unhelpful guarding of territory*” ([25], p. 1–2). Nor are we claiming that co-production should be considered the ‘gold standard’ or only way to approach participatory research – in any given situation, multiple factors will influence which participatory method is needed. Rather, we are attempting to highlight distinctive ethical and political features of co-production. These have tended to get diluted or lost altogether as the application of the term has expanded.

Equally, we do not claim there is or needs to be a single definition; not all co-production looks the same and it can be variously transformative or additive in its intent and effect [26]. While standardisation would belie this complexity, lack of standardisation does not legitimise labelling any or all forms of collaboration as ‘co-production’. The trend for doing so, what we term ‘cobiquity’, has led to co-production now being used to describe a “*fragmented set of activities, expectations and rationales*” ([27], p. 427). Despite this, there are key features and, particularly, values of co-production that are more generally considered common and essential. In making these features explicit, it becomes apparent that considering numerous and varied forms of research collaboration to be co-production risks losing sight of these critical qualities. In particular, the deliberate egalitarian rationale for co-production makes it stand out against other forms of collaboration or co-working. It is this aspect which we think is missing from the arguments put forth in the Oliver et al. Commentary [2].

Co-production, as understood in the expansive and diverse literature spanning from Ostrom [13] to Cahn [20] to Hickey et al. [23], offers a process whereby professionals and those traditionally on the receiving end of their ‘expertise’ (e.g. patients/service users/marginalised citizens) can collaborate with the goal of achieving outcomes that arguably cannot be achieved otherwise. It should engage the talents and experience of all involved and support the egalitarian relations and conditions needed to make the most of them. Logically, there is an expectation that these principles should apply to co-production in *research* (the focus of the Oliver et al. Commentary [2]) and that this means to some extent “*researchers giving up power and control they have inherited through its historical and structural distribution throughout the system*” ([28], p. 1275). Therefore, inherent to co-producing research is bringing together patients/service users/marginalised citizens with researchers and professionals/practitioners and attempting to form equitable partnerships. This extends to patients/service users/marginalised citizens making meaningful contributions to agenda-setting and the formation of research questions, not merely being ‘involved’ once these important decisions have been made by those who traditionally hold power in research settings. This draws otherwise excluded perspectives and understandings into strategic and procedural decision-making processes. That is to say, there is a clear egalitarian rationale supporting the co-production of research. Any research process labelled ‘co-production’ should therefore be assessed against the efforts made, and outcomes achieved, in alignment with these key features.

Oliver et al. [2] synthesise four main rationales for co-production from the literature – substantive, instrumental, normative and political. These can be subsumed within the two overarching rationales for patient and public involvement in health(care) outlined by Martin [29], namely technocratic (substantive, instrumental) and democratic (normative, political). This further refinement helps bring into focus the prioritisation throughout Oliver et al.’s [2] Commentary of the attainment of *technocratic* outcomes that uphold established research convention and benefit research careers, rather than the achievement of *democratic* values that benefit service users/patients/marginalised citizens. For example, they describe “*widespread advocacy*” for co-production as “*troubling*” on the basis that “*there is a significant dearth of empirical evidence about coproduction processes and outcomes*” ([2], p. 6). While evaluation can be useful, a democratic rationale does not require a sound evidence-base to justify the normative desirability of co-production. However, the Commentary gave short shrift to arguments more aligned with democratic rationales [30] – for example, addressing abuses of patient rights and related long-standing and enduring inequalities in power and influence between health(care) providers/researchers and patients/service users/citizens [31, 32].

Having referred to co-production as an “*exciting approach*” for health research, the authors caution that it “*should be done for the right reasons and in the right way*”. This begs the question: who decides what is right? We found very little in the Oliver et al. Commentary [2] to suggest that deciding what is ‘right’ in this context should be the task of anyone other than researchers or indeed their funders/employers. This is demonstrative of Oliver et al.’s [2] adoption of a technocratic approach to value which prioritises the specific needs of researchers and more broadly the perceived needs of research and science. This technocratic prioritisation is not explicitly stated in the Commentary but largely acts to define the considerations and recommendations within it. For instance, Oliver et al. [2] argue that co-production in research is more suitable when “*the policy or programme is widely regarded as a ‘good thing’ and the findings unlikely to be contested*”, and less needed when “*the nature and purpose of the policy or programme is relatively well defined and agreed upon*” (as outlined in Table 2, p. 7). In contrast, we argue that it is precisely these assumptions that should be laid open to challenge through processes of co-production.

## Costs of co-production or poor academic practice?

A full consideration of the tension and reciprocity between the technocratic and democratic rationales is beyond the scope of this paper. However, we outline this well-established distinction to highlight how, by neglecting democratic rationales for the co-production of research, Oliver et al. [2] perceive what we would describe as bad practice (i.e. ‘co-production’ that fails to adhere to the key features of this egalitarian tradition) as inherent flaws, or indeed the “*dark side*”, of co-production. The focus on technocratic value may account for the descriptions of ‘costs’ as static features that can be measured and assessed to determine whether co-production is ‘worth it’. The limitations this oversight imposes on the Commentary are evident in each of their core arguments about the “*challenges and costs*” of co-production (which we have adopted as sub-headings and responded to in the discussion below). By contrast, an egalitarian understanding asks who experiences these costs and what the alternative value could be of properly addressing them. Through this lens, we argue that each of the proposed challenges and costs can be understood as systemic or cultural barriers around power in research, which not only vulnerable partners (e.g. patients) but also researchers themselves can be detrimentally impacted by.

### Practical costs

The basis for this argument is that co-produced research “*is expensive, as it requires the presence or time of multiple actors who are often not on site, have other primary responsibilities, or need travel or other reimbursement*” ([2], p. 3). However, simply applying an economic logic would suggest co-production is relatively inexpensive when compared to international research collaborations and academic ‘networking’ activities. Likewise, in practice, the issue of ‘reimbursement’ is negligible in comparison to research staff costs, especially as in many instances patients/service users/citizens donate their time for free as their involvement is – unfortunately still – rarely sufficiently budgeted for by researchers.

The insufficiency of funding is emphasised by Oliver et al.’s adjoining observation that “*this type of work is often added on (to ‘real research’) with little thought for how to properly resource it*” ([2], p. 5). An economic critique of co-production should ask why it is that co-production is so rarely adequately considered or costed in research design. Such inquiry might offer greater insight into why co-production can be viewed as unequally burdening some researchers. Our view is that the low priority accorded to co-production in research is due to long-standing structural inequalities in academia. These have led to a disproportionate expectation/obligation on those who occupy less prestigious academic positions to carry out this type of work with insufficient resources – as suggested by Oliver et al. [2] and evidenced by a recent evaluation of community–university partnerships [33]. This is not a legitimate justification for dismissing co-production. Rather, it should lead researchers to be critical of the structural inequalities playing out in academia, which undermine both the importance of more participatory research approaches and the status and labour of those who undertake them.

Instead, Oliver et al. present a skill deficit in research as a failing of co-production; they argue that co-production requires interpersonal skills that “*not all academics are trained in or endowed with*” ([2], p. 5). Our experience is that the interpersonal skills that facilitate co-production do tend to be undervalued in research. However, this merely highlights a need for these skills to be better valued and developed in the research community, not a cost of co-production.

### Personal costs to researchers

The authors argue that “*inherent power imbalances and conflicts*” in co-produced research play out as interpersonal tensions, difficult conversations and/or outright disagreement ([2], p. 5). While we may want to avoid unconstructive conflict, difficult conversations or disagreements can be a welcome sign that different views, values, perspectives and experiences are being considered and discussed as part of a relational process. As Facer and Enright suggest, “*If it feels too easy, you probably aren’t doing it right*” ([33], p. 57); this is not to dismiss the “*emotional labour of working collaboratively*” ([2], p. 5) nor the challenges that will mean co-production is not always an entirely positive experience for all involved [34]. However, most research involves collaboration (albeit usually exclusively between researchers), and emotional labour, while unacknowledged, is intrinsically bound to such work. We argue that established hierarchies in academia and the relative power differences within research teams carry greater potential for emotional and career costs for researchers than co-production. Furthermore, in focusing on conflict, stress and burnout, the Commentary ignores the numerous examples where both researchers and ‘stakeholders’ find the process immensely rewarding (e.g. [35]). This is common in healthcare improvement research, where co-production provides the tools and opportunities to improve services that themselves cause stress and burnout. Again, it is important to acknowledge the cumulative effect of the structural inequalities that lead to the labour of co-production being unevenly distributed, i.e. overworked and often precariously employed ‘junior’ staff undertaking the challenge of co-production on top of everything else. Academic hierarchies, competing demands and precarity of employment exacerbate the potential for conflict, stress and burnout for such researchers; these should not be presented as hazards of co-production but rather as demonstrative of wider structural issues.

### Professional costs to researchers

Central to this argument is that co-production obstructs “*seeking research funding, writing publications in impactful journals, doing administration, and teaching*” and that this “*is a huge ask for academics”* ([2], p. 5). We encourage a more structural analysis that questions what has come to be seen as valuable within academia – why, for example, is publishing in high impact journals valued more highly than working directly with fellow citizens to improve society? Admittedly, achieving this within current institutional structures presents significant challenges but these are not conflicting values. If – as Oliver et al. [2] suggest – researchers who co-produce are regarded as “*light-weight*” within academia, and co-produced outputs considered “*lower quality*” and “*simply hard to publish*”, then we must challenge those assumptions and constraints. Indeed, why would researchers seek to maintain the status quo given widespread anxiety and discontent relating to the ever-increasing performance management and metricising of their contributions? Additionally, the notion that successful co-production requires researchers to “*engage with no expectation of a guaranteed impact as measured in academia*” ([2], p. 5) undoubtedly makes co-production extremely challenging but only if we accept that a narrowly defined output-focused culture is what academics should embrace.

### Costs to research

The authors point to delays caused by the additional work of recruiting and engaging with stakeholders. However, these activities only cause delays if research timetables do not reflect a thoroughly considered co-production approach. And of course, in this regard, recruitment to trials is hardly unproblematic [36]. The authors also fear that tensions later in the research process arising from differences in opinion on how to interpret findings and frame recommendations “*may result in research being co-opted or researchers being silenced*” ([2], p. 5). This is an example of where their argument might quite legitimately relate to inter-organisational collaboration but less so to co-production with patients/service users/marginalised citizens. In the latter context, claiming it is researchers who are in danger of being silenced seems naive given that one of the most pervasive critiques of participatory practice in healthcare/health research is that it commonly fails to represent and legitimise the voices and perspectives of service users and people from marginalised groups [22, 37–40]. Academics often have an implicit epistemology that privileges a certain hierarchy of knowledge and evidence (in research). Patients, service users and/or marginalised citizens who cannot speak the language of research are thereby silenced and their experiential, collective knowledge devalued and often excluded entirely [41–44]. This is a form of epistemic injustice [45]. In other words, when co-production fails to address pre-existing power dynamics in research it is service users and lay partners – not researchers – who are most likely silenced.

Additionally, such norms suggest researchers are uniquely qualified to determine research priorities without fully recognising that the translational gap between research and practice was/is largely due to researchers failing to conduct research with, and of relevance to, healthcare providers, policy-makers, patients and the public [46, 47]. Oliver et al. [2] argue that co-production can lead to “*dull*” and “*derivative*” research that is potentially damaging to academic careers. While it may not be their intention, their ambiguous framing of stakeholders leaves these statements open to the interpretation that they relate to the needs and preferences of service users, patients and marginalised citizens. This is a prime example of why transposing concerns regarding inter-organisational collaboration onto work with patients/service users/marginalised citizens is ill-advised. However, it also reflects the need for researchers and funders to show more interest in the everyday realities of people’s lives, rather than pushing them further to the margins of academic endeavour.

### Costs to stakeholders

Oliver et al. ([2], p.6) express concern for the vulnerability of stakeholders – “*especially policy-makers*” – who share sensitive information. However, looking outside the policy field, quality improvement research (and much else besides) is grounded in the identification of uncertainties [48], inefficiencies [49] and mistakes [50]. If we want research to enable improvement at broader institutional levels, then it is surely our role as researchers to surface such issues through ethical practices that protect our partners. Where is the value in defending a status quo where people cannot admit to feeling uncertain or making mistakes? Why not see creating safe spaces for professionals, patients, service users and citizens alike to experience and share vulnerability as something worth celebrating and promoting?

Batalden writes of the central role of sharing vulnerability in the building of trust and respect between patients and professionals through co-production; he argues “[b]*oth parties bring their knowledge, skill, and habits to the service making task. A willingness to be vulnerable arises from being fully present and able to fully engage another person*.” He explains that, while this “*idealised model does not always exist in practice*” it grants “*professionals important permission to be vulnerable and to value more fully the knowledge and skills patients bring to making health services*” ([51], p. 2). It seems illogical that Oliver et al. [2] perceive researchers to lack interpersonal skills and yet consider co-production problematic precisely because it puts professionals in positions that – in safe and supportive contexts – facilitate interpersonal development. In contrast, we argue for a need to create and evaluate contexts and supporting infrastructure to facilitate the building of trust and respect between researchers, professionals, patients, services users and citizens. The relative nature of vulnerability needs to be accounted for in the development of such environments as often the stakes are higher for those without professional prestige.

### Costs to the research profession

The closing argument is that working closely with parties with clearly vested interests risks transforming researchers into “*simply one more lobby group*” ([2], p. 6) as it has the potential to reduce trust in scientists and science. Often, such arguments come from those adopting a strict positivist position, which assumes the neutrality of researchers and views those with vested interests as invaliding the scientific endeavour. The claim implies that researchers’ own values and interests in participating in a research study are intrinsically of a different (moral?) order to those of other partners. We argue that research would benefit from reflection on all participants’ vested interests (including researchers’) and their influence on data collection and analysis. Additionally, the “*one more lobby group*” reference is a further example of where the authors transpose legitimate concerns about partnerships between researchers and practitioners to co-production between researchers and patients/service users/marginalised citizens. This transposition equates the egalitarian objectives of co-production – to create inclusive research practice capable of addressing the needs of patients/service users/marginalised citizens and society more broadly – with concerns as to the potentially corrupting role of, for example, industrial or political vested interests.

Overall, the thrust of this argument is that co-production promotes poor research practice and thereby undermines research. Our counterargument is that the tradition of co-production does not inherently promote poor practice; much the opposite – it gives greater consideration than is usual within academic research to issues such as representation, diversity, participation and dissemination. Conversely, there are many academic norms that lead to poor practice and should promote a mistrust in scientists and science, for example, the structural inequalities and organisational norms that result in the gendering of research hierarchies, disciplines, topics, participation and findings [52–54]. Wider adoption of the co-production tradition has the potential to help academia overcome the structural issues of exclusion, which pose a far greater threat to the credibility of science.

## What constitutes the dark side of co-production? Contemplating the research context

In acknowledging that Oliver et al. [2] have raised important questions in relation to co-production and agreeing with some of their broader points relating to partnership working, we hope this response is received in the spirit intended – to further the debate and dialogue around this important but contentious area. Here, we are responding to the imprecision of their definition of co-production, its neglect of the egalitarian imperative, and how this appears to have led them to select the wrong targets. Our argument is that this serves to make a scapegoat (co-production) for wider systematic failure (hierarchical and market-driven research environments). This blunts what may have otherwise been useful critique.

To clarify, we are not arguing for uncritical adoption of co-production – much the opposite. Even advocates of co-production acknowledge it is not always the most appropriate approach. Indeed, Ostrom herself clarified “*coproduction is not, of course, universally advantageous*” ([1], p. 1082). However, critique must come from a broader perspective than the one adopted by Oliver et al. [2]. It should take into consideration (1) the egalitarian rationale for co-production to further the rights and needs of patients, service users and marginalised citizens, (2) the wider structural issues of academia that impinge upon co-production in research, and (3) over 40 years of politically engaged research in this area. We are all involved in such research [3, 15, 28, 44, 55–62] and to that end, conducting work that Oliver et al. [2] appear to assume is currently absent.

We agree that doing co-production “*recklessly*” or “*discourteously*” has significant costs and that “*mindful engagement is essential for the ethical practice of research*” ([2], p. 6, 8). However, it is illegitimate to separate bad practice from the contextual factors facilitating and even promoting it, especially if such malpractice is then presented as the dark side of a process that has been misrepresented. The phenomenon of ‘cobiquity’ has diluted the legitimacy of both co-production and subsequent critiques of it, clouding inquiry and misdirecting what could otherwise be useful and therefore welcome scrutiny. Oliver et al. provide evidence of this when presenting examples of malpractice that are not inherent to the practice of co-production, nor in keeping with its egalitarian tradition. This is neither new nor helpful; we have already encountered researchers in the field using the Commentary as a rationale for not including patients, service users and the public in research design.

While one of us has argued elsewhere that co-production in the mental health field is currently an impossibility because of the very varied and entrenched power dynamics involved, especially for racialised groups [44], in contrast to Oliver et al. [2], this argument was not a call to minimise or avoid co-production. Rather, it was a call to attend to these issues so that real structural and social change might happen. Or – if this cannot happen – to increase scrutiny of practice labelled ‘co-production’ to highlight the limitations of practice that is largely disconnected from the egalitarian imperative. The Oliver et al. Commentary [2] merely highlights the value of ‘dark logic modelling’ more broadly – developing models to anticipate the most plausible unintended harmful impacts and associated mechanisms of health interventions and the need to guard against them from the outset of projects [63]. Quite simply, doing anything badly can be a costly risk.

Here, it is important to note that Oliver et al.’s Commentary [2] is by no means original in addressing the ‘dark side’ of co-production. Others writing of the ‘dark side’ of co-production – yet not cited in Oliver et al.'s Commentary – have described “*co-contamination*” [64] (an extension of the concept of “*co-destruction*” [65], which was presented as a logical counter to co-creation) and the “*seven evils*” of co-production [66], as well as emphasising the contextual distinction of “*co-production under the financial crisis*” [67]. For example, Steen et al. [66] raise the worrying trend of co-production projects being inequitable in design and appeal and consequently involving only already privileged population groups and by extension further marginalising others. Given the legitimacy of existing dark side critiques and our holding Oliver et al. in high regard for having written elsewhere of the potential for health interventions to have “*equity harms*” [68] and for suggesting radical shifts in health promotion for the furtherance of health equity [69], we were surprised that equity issues (beyond those concerning the division of collaborative research labour between researchers) were not more prominent in their delineation of co-production’s dark side.

Existing dark side critiques recognise that the *context* in which co-production occurs largely determines the nature of the process and outcomes. For instance, when Williams et al. assess what they term “*public value failure*” resulting from co-production (i.e. the dark side of co-production) they make it clear that the “*emphasis is not necessarily on highlighting the failure of co-production, but rather to shed light upon key factors that impede effective co-production processes*” ([64], p. 703). Steen et al. [66] even go so far as to explain one of the seven evils of co-production as how it can reinforce structural inequalities when used as a means of austerity to help governments abdicate their responsibilities to the public. For this reason, Fotaki warns against co-production “*instigating a race to the bottom*” ([67], p. 463). Similarly Moini argued that a certain “*technicalization of participation*” ([70], p. 158) works to shore up neoliberal regimes by creating the appearance of consensus and shared responsibility between different social actors and by offering technical solutions to what are essentially political problems (see also [19, 43]). Oliver et al. [2], however, do not offer a comparable critique. Instead, the arguments presented appear unaware of, or unconcerned by, how research predominantly provides a context for which co-production is ill-fitted. This is not, as suggested, because the *practice* of co-production is inherently flawed but rather because the current *context* and norms of research (or what they term “*business-as-usual rules*” ([2], p. 5) are corrupting. Their critique makes little reference to the relational spaces in research within which co-production occurs and overlooks essential dimensions of power. Where relationships have been mentioned, the reference is to researchers “*calling on favours and being able to provide favours in return*” and managing “*sometimes tense relationships*” ([2], p. 5) but the analysis does not explicitly address the power relations playing out in these instances. Doing so would highlight how the reality of inequitable power dynamics and deep-seated epistemological assumptions make co-production a highly political practice that must endeavour to transform traditional research processes as much as it does produce implementable outcomes. Oliver et al. [2] stray too close to ‘the problem’ of ‘co-production’ seeing only the dark side rather than what is casting the shadows.

## Conclusions: addressing ‘academic barriers’ to co-production and moving beyond technocratic priorities

We started our Commentary with Ostrom’s [1] quotation regarding ‘academic barriers’ preventing researchers from seeing greater potential in what can be achieved through co-production. We believe Oliver et al.’s Commentary to be an illustration of this; they argue co-production carries “*significant risks for academics*” because it requires them to “*adopt practices far from those traditionally taught, adopted, recognised or rewarded by the academy*” ([2], p. 3). This highlights that their primary concern with regards to the ‘costs’ of co-production is the need for researchers to protect their own interests (and careers) in what is an exclusionary system. Crucially, this is a status quo that is increasingly driven not by what they might construe as traditional values, but rather the principles of New Public Management, i.e. technocratic priorities, bureaucratisation and market fundamentalism [71, 72]. This concern in turn casts co-production as a corrupting force threatening the legitimacy of scientific endeavour and academic ‘achievement’. Their secondary concern appears to be how co-production can be shaped and used to help researchers play this competitive game better, thus allowing exclusionary academic convention driven by grant revenue generation and the need to create ‘impact case studies’ and internationally significant publications to fulfil national standards of ‘research excellence’ to continue unchanged. This misconstrues what we would characterise as the moral virtue of research and researchers as well as revealing how a “*culture of hit-and-run research*” ([2], p. 6) – driven by metrics and funder priorities that can often be disconnected from public value and egalitarian imperatives – perpetuates an instrumentalism that is unhelpful both to those outside such academic endeavours and, increasingly, to academics themselves.

Our main conclusion is that, in not addressing the structural factors that largely explain academia’s failure to accommodate and promote the egalitarian and utilitarian potential of co-produced research and instead focusing almost exclusively on technocratic utility, Oliver et al.’s Commentary [2] serves to reinforce the foundations of the status quo. Interestingly, Oliver et al. argue “*political reasons for engaging in coproductive research may be the least-discussed, yet most important rationale made by researchers*” ([2], p. 8). This appears to illuminate a blind spot in the authors’ argument. Far from being “*the least discussed*”, the emancipatory politics of co-production are explicitly and consistently articulated by those who advocate this practice and we contribute this Commentary to that literature [19–21, 30, 73–75]. Additionally, it seems inconsistent for Oliver et al. [2] to make this statement without also explicitly discussing their own rationale. We argue it is overly technocratic and, consequently, while the Commentary is presented as apolitical, it leaves unacknowledged the significant opportunity costs and harms attributable to researchers neglecting egalitarian rationales. The significance of this is that their Commentary serves to make health research less inclusive and further removed from the public it should be serving. Therefore, Oliver et al. [2] provide a useful reminder that, when adopting dark logic, it is important not to get lost in the shadows.

## Data Availability

Not applicable.

## Abbreviation

NIHR CLAHRCs: National Institute for Health Research Collaborations for Leadership in Applied Health Research and Care
